# Supplementary material for: Effects of Ultra-Processed Diets on Adiposity, Gut Barrier Integrity, Inflammation, and Microbiota in Male and Female Mice
Source: Nutrients. 2025 Sep 30;17(19):3116. doi: 10.3390/nu17193116 (PMC12526148; doi:10.3390/nu17193116)
Supplement: Supplementary file 1 [file nutrients-17-03116-s001.zip › Supplementary Figure S1.pdf]

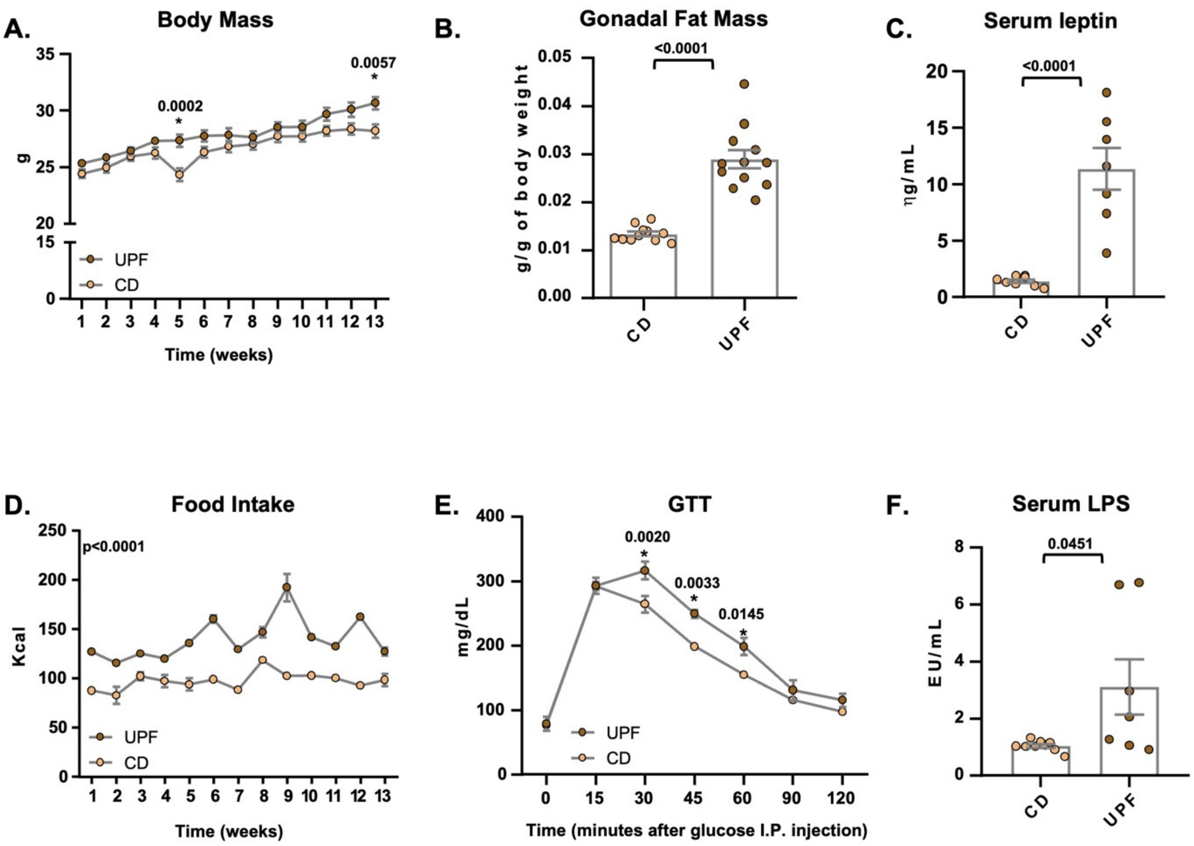

Figure S1. UPF diet increases body mass, adiposity, serum leptin, and metabolic alterations. Male mice received a chow diet (CD) or an ultra-processed food diet (UPF) for 13 weeks. Body mass (**A**), n=8 CD and 7 UPF. Gonadal fat mass (**B**), n=11 CD and 12 UPF. Serum leptin (**C**), n=8 CD and 7 UPF. Food intake (**D**), n=11 CD and 12 UPF. Glucose tolerance test (GTT) (**E**), n=6 each group. Serum LPS (**F**), n=8 CD and 7 UPF. Data are mean ± SEM. Statistical analyses were performed using two-way ANOVA with Bonferroni's post hoc test, except for Panels (B, C, F), where two-tailed unpaired t-test was applied. $p < 0.05$ was considered significant and is shown in the figure.
