# Supplementary material for: Effects of Ultra-Processed Diets on Adiposity, Gut Barrier Integrity, Inflammation, and Microbiota in Male and Female Mice
Source: Nutrients. 2025 Sep 30;17(19):3116. doi: 10.3390/nu17193116 (PMC12526148; doi:10.3390/nu17193116)
Supplement: Supplementary file 1 [file nutrients-17-03116-s001.zip › Supplementary Table S1.pdf]

	Nuvilab CR1® (100g)	UPF Pellet (100 g)	Snack (100g)	Cake (100g)
Energy content (kcal)	336	467	559	410
Carbohydrates (g)	53	49	55	55
Proteins (g)	22	17	5.1	6.2
Lipids (g)	4	26	35	18
Dietary Fibers (g)	7	5.7	2.8	1.4
Sodium (mg)	270	259	602	283
Vitamin A (µg)	765	301	7.5	
Vitamin D (µg)	10	3.8		
Vitamin E (mg)	5.5	2.2	2.9	
Vitamin B1 (mg)	1	0.55	0.43	0.13
Vitamin B2 (mg)	1.3	0.61	0.29	0.09
Vitamin B6 (mg)	0.02	0.1	0.14	0.05
Vitamin B12 (µg)	4	1.7		
Calcium (mg)	1.3	64	1.3	75
Iron (mg)	18	7.9	18	2.1
Zinc (mg)	11	5.4	11	0.71

Source: IBGE and TACO – Brazilian Food Composition Tables.
